# Hydro-Thermal Fatigue of Polymer Matrix Composite Biomaterials

**DOI:** 10.3390/ma12223650

**Published:** 2019-11-06

**Authors:** Daniel Pieniak, Krzysztof Przystupa, Agata Walczak, Agata M. Niewczas, Aneta Krzyzak, Grzegorz Bartnik, Leszek Gil, Paweł Lonkwic

**Affiliations:** 1 Department of Mechanics and Machine Building, University of Economics and Innovations in Lublin, Projektowa 4, 20–209 Lublin, Poland; daniel.pieniak@wsei.lublin.pl (D.P.); grzegorz.bartnik@wsei.lublin.pl (G.B.); leszek.gil@wsei.lublin.pl (L.G.); 2 Department of Automation, Lublin University of Technology, Nadbystrzycka 36, 20–618 Lublin, Poland; 3 Faculty of Safety Engineering and Civil Protection, The Main School of Fire Service, Slowackiego 52/54, 01–629 Warsaw, Poland; awalczak@sgsp.edu.pl; 4 Department of Conservative Dentistry with Endodontics, Medical University of Lublin, Karmelicka 7, 20–080 Lublin, Poland; agata.niewczas@umlub.pl; 5 Faculty of Aeronautics, Military University of Aviation, 35 Dywizjonu 303, 08–521 Deblin, Poland; a.krzyzak@law.mil.pl; 6 The Institute of Technical Sciences and Aviation, The State School of Higher Education, Pocztowa 54, 22–100 Chełm, Poland; plonkwic@pwsz.chelm.pl

**Keywords:** wear, hardenss, surface layer, thermocycling, dental materials

## Abstract

This study discusses a quantitative fatigue evaluation of polymer–ceramic composites for dental restorations, i.e., commercial (Filtek Z550) and experimental Ex-nano (G), Ex-flow (G). Their evaluation is based on the following descriptors: mechanical strength, elastic modulus and strain work to fracture. Supposed to reflect factors of environmental degradation conditions, thermal fatigue was simulated with a special computer-controlled device performing algorithms of thermocycling. The specimens intended for the strength test underwent 10^4^ hydro-thermal fatigue cycles. This procedure of thermocycling was preceded by aging, which meant immersing the specimens in artificial saliva at 37 °C for 30 days. The strength tests after aging only and after aging and thermocycles were performed in line with the three-point flexural strength (TFS) test, specified in ISO 4049, and the biaxial flexural strength (BFS) test, specifically piston-on-three-ball in accordance with ISO 6872. Based on the results, it can be stated that composites with higher volume content of inorganic particles after aging only show higher strength than materials with lower filler particle content. For example, the average flexural bending strength of the Ex-flow (G) composite was about 45% lower than the value obtained for the Ex-nano (G) material. The residual strength after thermocycles is significantly lower for the experimental composites, whereas a smaller decrease in strength is recorded for the commercial composites. Decreases in strength were about 4% (Filtek Z550), 43% (Ex-nano (G)), and 29% (Ex-flow (G)) for the BFS test; and about 17% (Filtek Z550), 55% (Ex-nano (G)), 60% (Ex-flow (G)) for the TFS test. The elastic modulus of the experimental composites after only aging is higher (about 42%) than that of the commercial composite, but the elastic modulus of the commercial composite increases significantly after thermocycling. A descriptor known as strain work to fracture turns out to be a good descriptor for evaluating the hydro-thermal fatigue of the tested polymer–ceramic composites.

## 1. Introduction

Composite materials are used in a variety of applications due to their special properties and benefits for transferring loads in biomechanical systems [1,2,3,4,5]. Composite biomaterials are presently common in dental practice. Polymer–ceramic composites, more precisely light-cured polymer matrix ceramic composites (LC PMCCs), are mainly applied as fillings in human teeth, but are also applied in structures for permanent composite dental prostheses like crowns and bridges, dentures, and partial dentures, as well as temporary and permanent splinting [5,6].

Their mechanical properties, which are important in the operational environment, depend on various factors, such as the materials’ microstructure, failure mechanisms and environmental effects [7,8,9]. When considering the microstructure of a composite, crucial aspects include: the type and content of the resin matrix; the filler particle size, content and dispersion; and the adhesion between the matrix and the filler [10,11]. Therefore, all new LC PMCCs should be tested in order to determine their properties.

In the mouth environment, dental composites undergo cyclic occlusive and thermal loading, so their high fatigue resistance is a fundamental factor in their design [12]. Here, the authors focus on thermal fatigue and related aging/hydrolitic fatigue, denoted in [13] as hydro-thermal fatigue. 

Thermal fatigue chiefly results from mechanical deformation in the material by acting bonds. These bonds can be classified as internal or external [14]. External bonds include all elements that restrict the freedom of deformation of the material. Internal bonds can result from temperature gradient, anisotropic structure, and phases with different coefficients of thermal expansion. In polymer–ceramic composites, the filler and the matrix have different coefficients of thermal expansion, e.g., the linear mean value of this parameter for composite resins in an operating temperature range of 0–60 °C is as follows: Bis-GMA—120.3⋅10–6/°C; TEGDMA—110.1⋅10–6/°C; whereas for the microhybrid composite Filtek Z-250, it is 33.0⋅10–6/°C. The process of thermal fatigue is regarded as a consequence of a synergetic operation of two processes, i.e., cyclical deformation and creep [15]. Additionally, stress relaxation occurs at the highest temperatures of the cycle, in heating. Experiments confirm thermal stress relaxation in polymer composites [16] and that the speed of these processes is also important in cyclic loads. Milewski [17] claims that the capability of the material to relax local fields of stress affects its fatigue strength. Also, [18] proves that processes of creep and relaxation depend on the filler content and the composition of the monomer, as well as the shape and size of the filler particles. It has been shown that hybrid composites with more differentiated particle shapes and sizes are more resistant to higher fatigue damage [19]. 

It is obvious that hydrolytic aging of polymer–ceramic composites occurs, but destruction mechanisms have not yet been clearly identified. Researchers believe that water in a polymer matrix composite structure acts like a plasticizer, so stress is relaxed and stiffness is reduced [20]. It is also probable that chemical compounds decompose the polymer network in the aqueous solution by disintegrating ester bonds [21]. It is also considered that moisture absorption depends on the number and type of filler particles, as well as the type of monomers used in the matrix production [22]. Thermal diffusion of water molecules can occur in the process of hydro-thermal fatigue, which can easily be seen in the swelling and contracting of wood-based materials, polymers and concrete [14]. This type of diffusion is irreversible.

In state-of-the-art applications, hydro-thermal fatigue depends to a large extent on microstructural changes; more precisely, on damages in a zone of organic (resin) and inorganic (filler) phase separation caused by acting local structural bonds. Local thermal deformations of phases are different, contributing to interphase stresses, whereas anisothermal cyclic thermal interaction causes a stress gradient. Variability in temperature fields related to an impact of moisture is probably more destructive. With reference to the above considerations, it can be assumed that hydro-thermal fatigue of polymer–ceramic composites leads to an unfavorable change in their functional properties, including mechanical properties, which are clinically fundamental.

The aim of this study was to determine the mechanical properties of new, experimental composites and to compare the results with commercial ones under operational conditions. More precisely, the study aims to quantitatively evaluate the fatigue strength of selected polymer–ceramic composites by means of descriptors like mechanical strength, elastic modulus and work to fracture.

## 2. Material and Research Method

### 2.1. Materials

**Figure 1:**
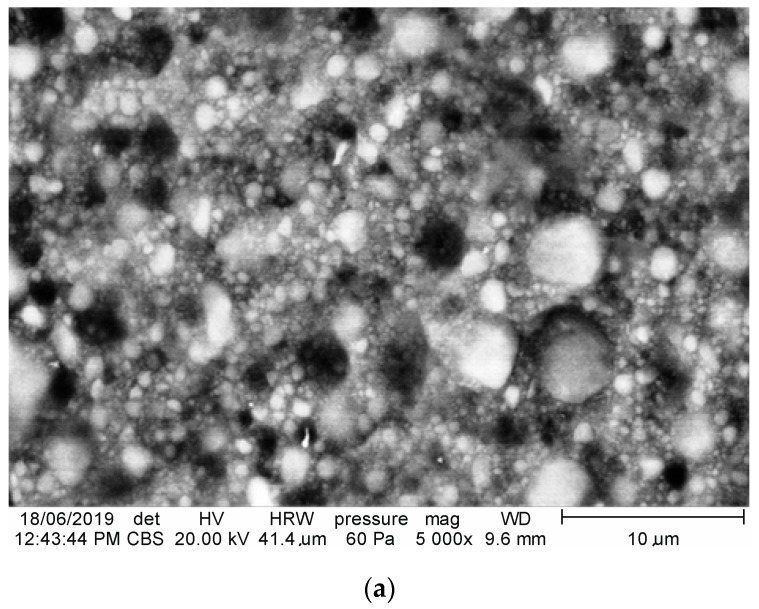

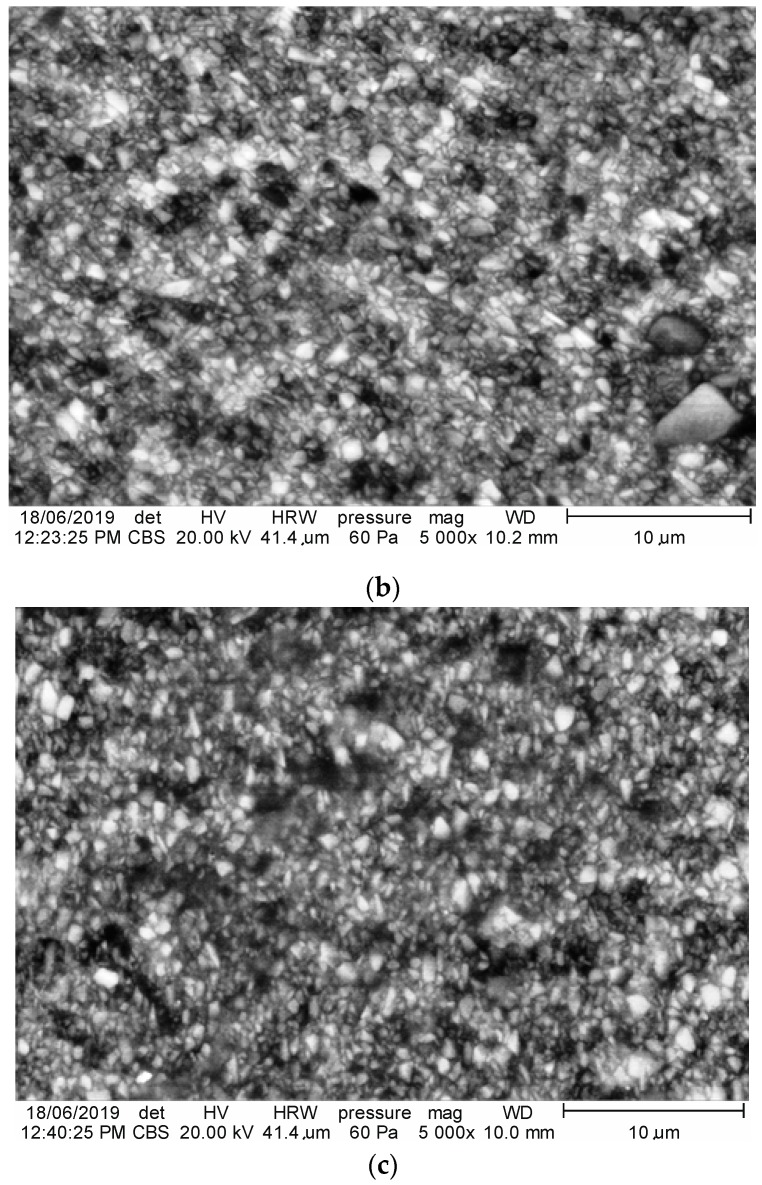
SEM microimages of the fractured surfaces of the composite structures: (**a**) Z550, (**b**) Ex-nano (G), (**c**) Ex-flow (G).

In this study, three groups of materials—two experimental and one commercial (referential)—were selected: Ex-nano (G), Ex-flow (G), and Filtek Z550 (3M ESPE). These materials are identified in Table 1. Specimens of these composites were prepared in accordance with the manufacturers’ recommendations. This means that the specimens were shaped by a single operator in a metal split-mold and then light-cured using Megalux LED (Megadenta, Radeberg, Germany) 1200 mW/cm^2^ for 40 s with a soft-start system. The specimens were polished after polymerization with abrasive discs covered with a layer of diamond particles in a resin matrix of 600 and 1200 grid on a single wheel grinder and polisher Saphir 550 (ATM Gmbh, Mammelzen, Germany) equipment and then cleaned in water. Morphologic images were made by scanning electron microscopy (Quanta FEG 650). SEM (FEI, Hillsboro, OH, USA) microimages of morphology of the composites are given in Figure 1. These images were observed in the break faces after strength tests.

### 2.2. Method of Thermocycling 

Multi-component materials based on polymers are sensitive to cyclic temperature fluctuations. Some authors report that in the mouth environment, there are 6000 thermal cycles in five years [23]. This process can lead to thermal fatigue of polymer–ceramic composites [6].

The simulated thermal fatigue process under laboratory conditions is known as thermocycling. Thermocycling simulates real temperature fluctuations corresponding to physiological processes, i.e., eating, drinking, breathing [24,25]. Results achieved in such a way cannot be clearly interpreted with respect to clinical conditions. In real (clinical) conditions, there are also differences between patients because of, e.g., their dietary habits [26]. Thermocycling, however, offers high approximation and is frequently applied by researchers [27,28,29]. 

Prior to our experiments, all of the specimens were immersed in artificial saliva at 37 °C for 30 days to simulate an aging process. Then, half of them underwent strength tests and the second half underwent thermocycling. In this study, the groups were formed according to the type of material as the aging only procedure (control group) and the aging and thermocycling (TC) procedure (n = 10). All thermocycles were conducted between 5 °C and 55 °C for a dwell time of 30 s. Time of filling and emptying the vessel with a working liquid (water) was 15 s. The number of thermal cycles was 10^4^, which according to the review article [30] is considered to be a sufficient number. 

### 2.3. Methods for Investigating Strength 

**Figure 2:**
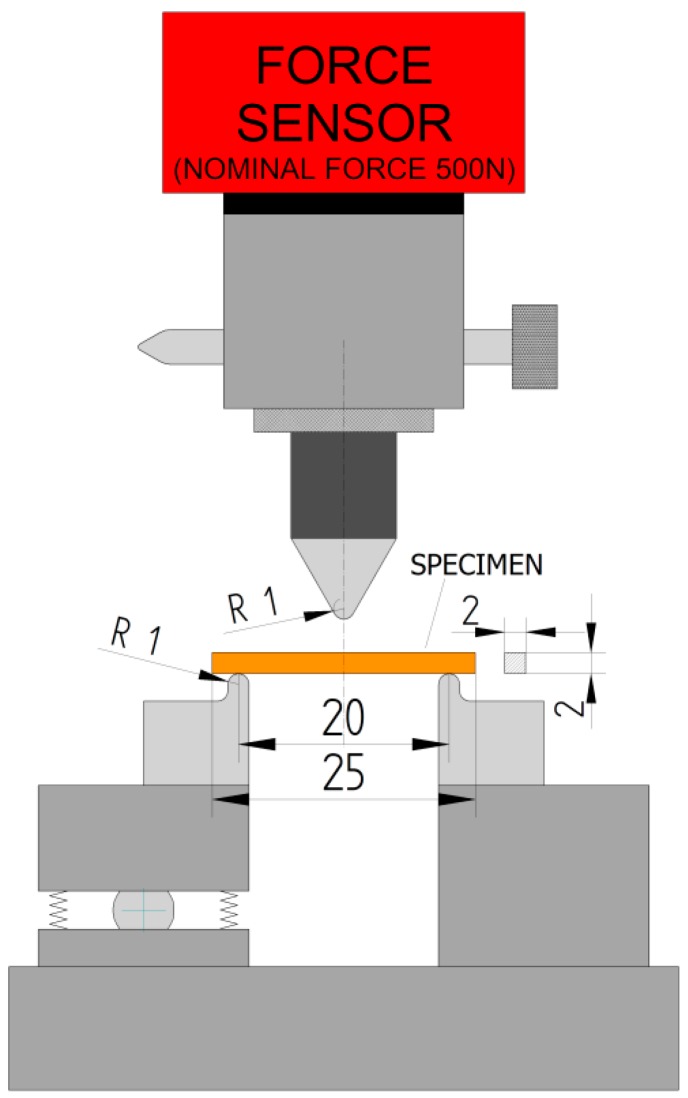
Support system, pin and specimen in the three-point bending test (values are given in millimeters).

Two types of strength tests were performed. The bending strength test was a three-point bending test that does not entirely reflect the loads that occur in clinical applications [31]. This is, however, a method codified by ISO 4049 [32], and tests conducted in line with these rules can be easily compared. Twelve specimens per material group of dimensions *b =* 2 mm (width)*, d =* 2 mm (thickness)*, c =* 25 mm (length) were prepared (Figure 2). The strength test was performed with the Zwick/Roell Z100 (ZwickRoell GmbH & Co. KG, Ulm, Germany) with a traverse speed of 0.5 mm/min, support span *L =* 20 mm, and radius of the supports and pin are 1 mm (Figure 2). In tests, a nominal force applied with the use of Xforce load cell was 500 N.

According to ISO 4049 [33] on dental composite materials applied to reconstruct hard dental tissues, the strength of the material is chiefly specified by the three-point flexural strength (TFS) test [34]. The literature, however, describes the application of other research methods, e.g., the biaxial flexural strength (BFS) test [35,36,37].

**Figure 3:**
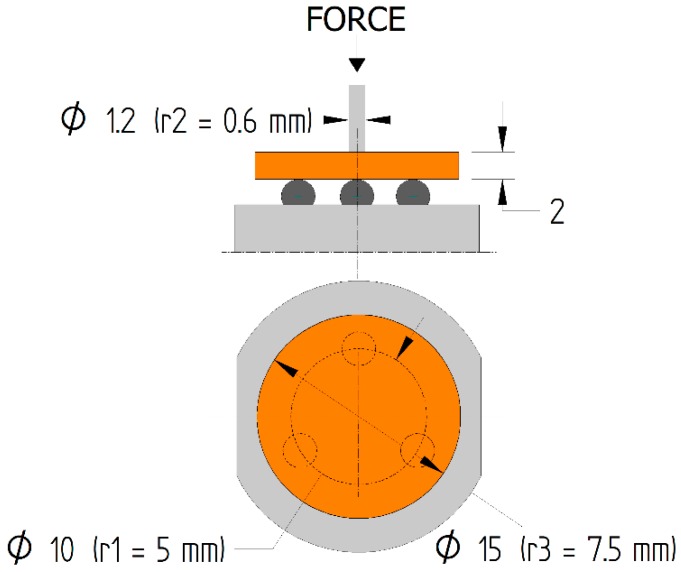
Schematics of the location of the specimen in the BFS test—front and top views (values are given in millimeters).

Covered by ISO 6872 [38], the BFS test means that maximum tensile stresses occur in the central part of the specimen and decrease by dispersing over a larger area, or by increasing the radius between the area of tensile stress and the center of the specimen [33]. A positive aspect of this method is that rounded specimens are used. Maximum stresses in the center of the specimen prevent cracks at edges that affect specimen strength. It should be emphasized that the zone of stress is smaller than that in the TFS test, so stresses in the defective volume are less likely to occur. It should be added that the process of polymerization of rounded specimens is more repeatable [34]. In the BFS test (manufacturer, city, country), the fifteen specimens per material group were used, which were in the shape of discs with a thickness of 2 mm thick and a diameter of 15 mm. The BFS test is schematically shown in Figure 3.

The BFS test made it possible to determine the tensile strength σ_BI_ in accordance with the following formulas:

(1) $$\sigma_{BI}=\frac{-0.2387\;P\left(X-Y\right)}{h^{2}}$$

(2) $$X=\left(1+\nu \right)\;ln{\left(\frac{r_{2}}{r_{3}}\right)}^{2}+\left[\frac{1-\nu }{2}\right]{\left(\frac{r_{2}}{r_{3}}\right)}^{2}$$

(3) $$Y=\left(1+\nu \right)\left[1+ln{\left(\frac{r_{1}}{r_{3}}\right)}^{2}\right]+\left(1-\nu \right){\left(\frac{r_{1}}{r_{3}}\right)}^{2}$$

where:

*P*—maximum force, given in N,
*h*—specimen height, given in mm (Figure 3),
*ν*—Poisson ratio, for of the tested composites is ν = 0.24 [34]
*r_1_*—circle radius of the steel rounded grips (Figure 3), r_1_ = 5 mm,
*r_2_*—pin surface radius, r_2_ = 0.6 mm,
*r_3_*—specimen radius, given in mm (Figure 3).

## 3. Results and Discussion

### 3.1. BFS and TFS Strength (Aging Only)

**Figure 4:**
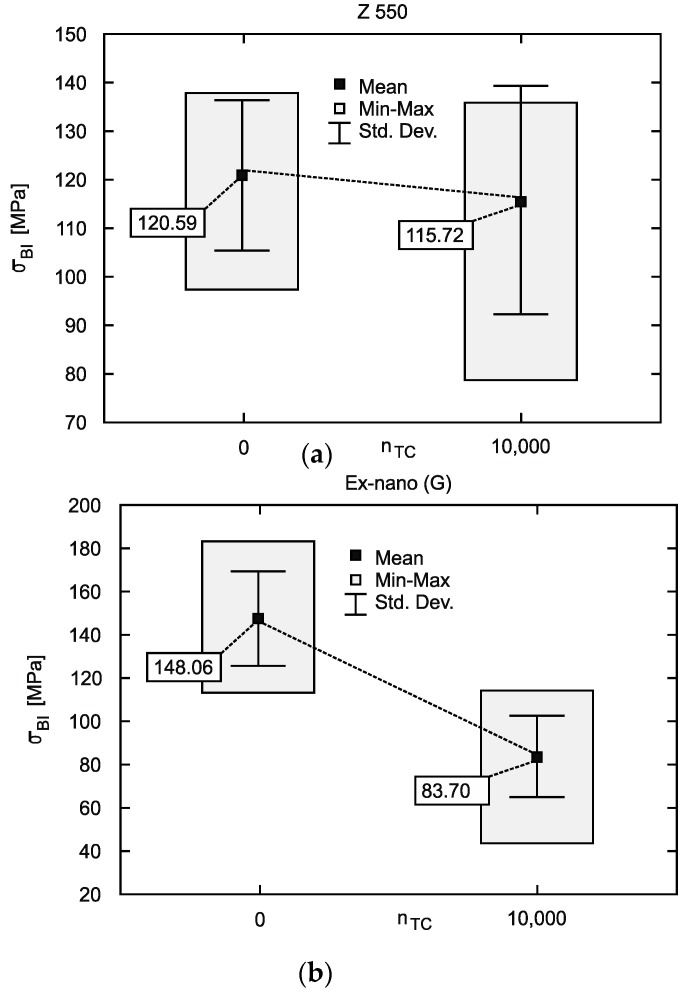

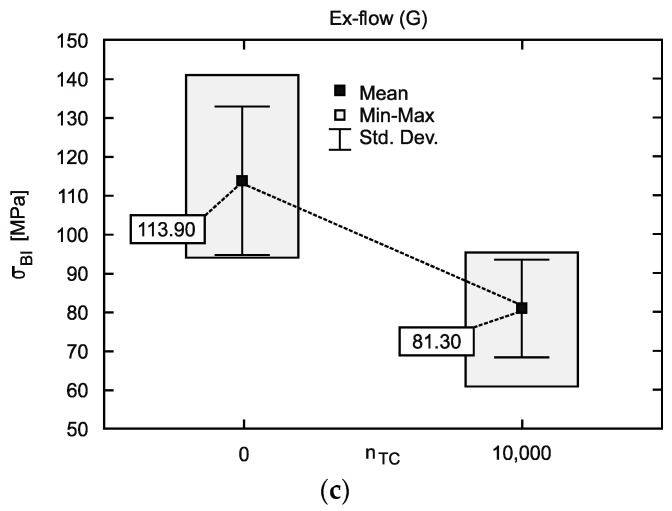
Tensile strength in the BFS test (**a**) Filtek Z550 composite, (**b**) Ex-nano (G) composite, (**c**) Ex-flow (G) composite.

The results of the BFS test with the following quantities are given in Table 2: F_BI_—maximum force, dL (F_BI_)—specimen’s deflection under the maximum force, F_Fract_—damage force, dL(F_Fract_)—maximum deflection, W(F_BI_)—strain work to maximum force, W(F_Fract_)—strain work to fracture, *n_TC_*—number of conducted hydro-thermal cycles, $\bar{x}$—mean, s—standard deviation, CV—coefficient of variation. The mean values and standard deviation of the biaxial flexural strength (σ_BI_) are given in Figure 4. Table 3 presents the results of the TFS test with the following quantities: E_f_—flexural modulus, σ_fm_—bending strength, ε_fm_—specimen’s deflection under the maximum force, σ_fB_—damage stress, ε_fB_—specimen’s deflection in damage, W_fm_—strain work to maximum force, W_fB_—strain work to fracture. 

The Ex-nano (G) achieved the highest strength in the BFS and TFS tests after aging in artificial saliva (Figure 4b and Table 3). This material contains the smallest average number of filler particles, whereas the filler content by weight is the same as in the Z550 material and higher than in the Ex-flow (G) composite. As predicted, the BFS strength of the Ex-flow (G) composite with the lowest filler content was the lowest among the tested materials (Figure 4c). This relationship is in agreement with other studies [16,39,40,41]. On the other hand, some authors claim that there is no relation between the filler content and strength of composites [42,43]. In the paper, the influence of the filler size on strength can only be seen in the case of the TFS test. It seems that this is connected with the occurrence of the failure mechanism in the specimen during these two tests. Some authors observed no effect of filler particle size on strength [44,45]. Similar relationships as for tensile strength were obtained in our own research when the W(F_Fract_) parameter was analyzed (Table 3), which is consistent with the literature reports, because bending work depends exponentially on the filler content [46]. Increasing the filler content, similarly as for the strength parameter, increases strain work to fracture but to a certain level only (critical concentration), above which this property improves only slightly [16,33], and sometimes even deteriorates [47].

The normative requirements for mechanical strength in ISO 4049 define the TFS bending strength only. Specified in this ISO 4049 standard, the value of TFS strength for the composites tested in this paper is ≥ 80 MPa. Z550 and Ex-flow (G) materials aged in artificial saliva for 30 days at 37 °C did not show the appropriate minimum TFS strength (Table 3). It should be pointed out that in the TFS test, the exposed side of the specimen on the supporting structure faced up (Figure 2) towards the strain, which, as the authors of this paper claim, better reflects real conditions of use, but may influence the achievement of lower strength, namely, in the general theory on material strength, the largest stress was demonstrated to occur in the upper and lower layers of the beam material, whereas stress is nearly zero in the neutral layer passing through the center of gravity of the cross-section. The lower layer, which in this study is on the unexposed side, is stretched. It is possible that despite the fact that material manufacturers allow even 3 mm curing depth of composites (ISO 4049 specifies that the curing depth of a single layer should not be less than 1.5 mm), the composite on the exposed side was more strengthened. Moreover, the compression strength of RBCs falls within the range of 200–400 MPa and the tensile strength within 30–60 MPa (from the diametral tensile strength test) [48], which indirectly indicates that the stretched beam layer is more vulnerable to destruction initiation. 

The literature, e.g., the paper [49], specifies the recommended minimum value of elastic modulus as ≥ 10 GPa (10^4^ MPa). Determined in the TFS test, the values of the elastic modulus after aging of the Ex-nano (G) and Ex-flow (G) materials were similar to the expected value given in the literature. The elastic modulus after aging of the Z550 material was, however, lower than expected (Table 3). Nevertheless, more generally, the elastic modulus of RBCs can be 3 ÷ 15 GPa [50,51]. The lower value of the elastic modulus of the Z550 may be caused by the size of filler particles. According to Ilie, there is a relationship between the filler particle size and the elasticity modulus of the composite [48]. Moreover, the lower value may result from, e.g., moisture absorption into the composite structure in the aging procedure. Moisture sorption into a matrix of nano and nano-hybrid composites is ~ 5 ÷ 7.5% after immersion in the liquid for 30 days at a temperature ~ 37 °C [52]. The moisture content of the specimens was not measured in the present study. The section surface areas of the specimens used in the TFS after the aging procedure were, however, different due to the type of material. All of the specimens were made in the same manner and form, and they later underwent the 30-day aging procedure in artificial saliva. The average cross-section areas of the Z550, Ex-nano (G) and Ex-flow (G) after aging were: 5.13 mm^2^, 4.42 mm^2^ and 4.41 mm^2^. The cross-sectional area of the Z550 was the largest, which may indicate that the specimens of this material swelled more than those of the experimental materials. According to [20], moisture in the structure of a polymeric matrix composite acts as a plasticizer, and its action leads to relaxation of natural stresses and reduction of stiffness, which probably influenced the average elastic modulus of the Z550 material under these specific conditions (Table 3).

### 3.2. Impact of Thermocycling

The influence of thermal cycles on the deterioration of the resistance to damage was demonstrated in [53], and a decrease in bending strength of composites subjected to thermal shocks was recorded in [22]. These authors first immersed the materials in water for 30 days at a temperature of 37 °C and then subjected them to thermal loads (temperature range: 5–55 °C; number of cycles: 500; exposure time: 30 s). The specimens after such thermal loading conditions were compared to those immersed in water for 24 h at a temperature of 37 °C. A decrease in strength of the tested composites ranged from ~1.4% to 44%. The influence of thermocycling on the bending strength of LC PMCCs was investigated by [25] under the following conditions: cycle temperature range: 5–55 °C, exposure time: 30 s and number of thermal load cycles: 0 cycles (the material was immersed in water for 24 h), 15,000 cycles, 30,000 cycles and 45,000 cycles. The investigation showed a significant decrease in bending strength after 15,000 cycles for one material only, whereas a large strength decrease occurred for the other materials after 30,000–45,000 cycles. The experiments [54] showed a high decrease in bending strength of all the tested materials after 5000 cycles of thermal loads (temperature range: 4–60 °C, exposure time: 60 s). A decrease in strength ranged from 31% to 41%, whereas the strength achieved between 5000 and 20,000 cycles remained at a similar level. Micro-cracks between filler particles and the matrix did not occur in materials after 5000 cycles [54]. No significant influence of thermal loads on the bending strength of materials was recorded [55] (temperature range: 5–60 °C, number of cycles: 100,000, exposure time: 15 s). The authors claim that the maximum temperature was relatively low (max. 55 °C) and did not significantly degrade the material [56]. On the other hand, a temperature of 60 °C could have contributed to the initiation of secondary polymerization, and consequently to the improvement of mechanical properties [57]. To sum up, the above-mentioned research results cannot give a satisfactory answer to the question of the impact of thermal cycles on the functional properties of PMCCs, and do not allow for drawing general conclusions. In light of the findings reported in the literature, the impact of thermal cycles of inherent parameters and numbers on each material should be determined independently. 

**Figure 5:**
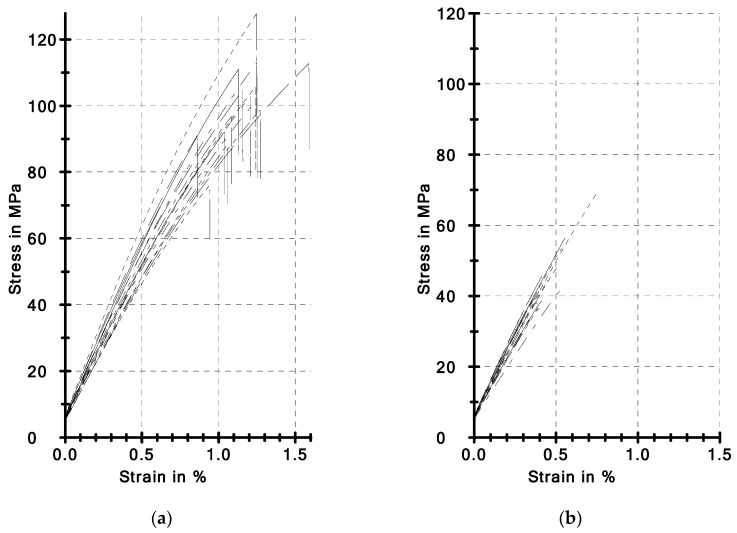
Comparison of *σ*-*ε* characteristics obtained in the TFS test: (**a**) Ex-nano (G), (**b**) Ex-nano (G) after 10^4^ thermocycles.

In our research, the decrease in strength was dependent on the type of material and strength test. The bending strengths in the TFS test after 10^4^ TC for the Z550, Ex-nano (G) and Ex-flow (G) decreased by 16.57%, 55.38% and 60.48%, respectively; whereas the tensile strengths in the BFS test were 4.32%, 43.47% and 28.62%, respectively. The degree of degradation of strength properties is varied. The mechanism of destruction is possibly also different, because PMCCs under the influence of hydro-thermal shocks underwent thermal and hydrolytic degradation, which was also confirmed [58]. Thermal degradation usually manifests itself in the formation and propagation of micro cracks in the material [54,59], which often occurs at the composite phase boundary [26,60]. Micro cracks due to the varied temperature field are favored by significant differences in thermal expansion of composite components. Micro cracks facilitate the formation and propagation of macro cracks in the specimen, which is also confirmed by the *σ-ε* characteristics in Figure 5. The Ex-nano (G) specimens not subjected to thermal shock show elastic-plastic properties, and are destroyed at a higher value of stress and deformation (deflection) than the specimens after thermocycling. On the other hand, after thermal shocks, macro damage is initiated at lower values of stress and deformation; destruction of all of the specimens after TC was catastrophic, without a breaking down phase. The impact of the viscoelastic behavior of the polymer phase after TC was also very limited.

Hydrolytic degradation may have a significant impact on the deterioration of strength properties of the studied polymer–ceramic composites in comparison with similar resin-based composites. This problem has been described in several papers [20,22,61,62]. It was shown that the resistance of PMCCs to cracking increases during the initial stages of exposure in humid environments, but that during the following stages of such exposure, ΔKth (threshold value of fracture toughness K_c_) significantly decreases, which can partly explain our research results. The degradation of the composite is more related to damage at the phase boundary than to hydrolytic degradation of the matrix [16]. In the nanocomposites investigated in our research, the key factor may be the damage of the so-called interphase. The interphase is a kind of zone connecting composite phases, where the forces between the matrix and reinforcement are redistributed. The range of mutual interaction between the matrix and reinforcement differs and depends on the forces of chemical bonds or only weak friction forces between phases. The interface in PMCCs is usually 2–9 nm thick [63]. The quality of the interphase can be indirectly evaluated in microscopic observations of fractured surfaces. It was found that the remaining resin layer on the surface of filler particles suggests that the crack propagated not at the filler particle border, but in the resin phase [16]. Such a manner of destruction can be explained by residual stresses created in photopolymerization of resin composites and acting in the interphase. These stresses cause a deviation of progressive crack from the interphase, which indirectly proves a good quality of the interphase. Microscopic observations of fractured surfaces in the experimental composite investigated in our research (Figure 1) do not confirm that resin layers remained on the filler particles. Hydro-thermal fatigue seems to adversely affect the interphase of the experimental composites, whereas the degradation of the interphase in the Z550 material was limited. While producing the Z550 composite, the complicated process of silanization of filler particles, which turned out to be effective, is probably applied. During curing, silane-coated particles chemically combine with the polymer matrix to form the interphase [64]. The effect of silanization seems to last much longer in the case of nearly spherical filler particles, and such a filler was used in the Z550 composite. 

The elastic modulus of the Z550 material significantly increased after thermocycling (Table 3), which could mean that the maximum temperature of the thermal cycle, set as 55 °C, could cause phase transitions leading to increased stiffness. The glass transition temperature (T_g_) of the Filtek Z250 (one of the early generations of the Z550 composite, the microhybrid material, produced by the same manufacturer; components of the resin matrix are similar to those in the Z550 material) in humid environments is ~ 45 °C; Bis-GMA (one of the main components of the composite’s resin matrix) is ~ 37 °C; and the wet structures that absorb moisture are ~ 22 °C and 38.7 °C, respectively [65]. Therefore, it is possible that secondary polymerization due to thermal treatment increased the stiffness of the Z550 composite structure. The question is why the strength of the Z550 did not increase like the elastic modulus. Belzowski explains that the values of elasticity coefficients represent the average state of the material structure with load history, and the remaining strength depends on damage causing the largest local weakness [66]. Therefore, these two quantities are not mutually representative as descriptors of fatigue degradation.

## 4. Conclusions

Within the limits of the study, it can be stated that the process of hydrothermal fatigue degradation of LC PMCCs varies, so it is necessary to test all newly designed materials. It was indicated that the experimental composites, i.e., Ex-nano (G) and Ex-flow (G), are less durable than the commercial composite Z550 due to hydro-thermal fatigue. Deterioration of strength due to thermocycling of experimental composites was higher than a commercial one. It seems that the high initial strength of PMCCs does not always contribute to resistance to hydrothermal fatigue. On the other hand, thermocycles cause higher changes in elastic modulus in the case of the commercial composite compared with experimental materials. It should also be highlighted that values of elastic modulus were higher for the Ex-nano (G) and the Ex-flow (G) than the Z550 material. 

Most phenomenological hypotheses on accumulation of fatigue damage described in the literature are based on an evaluation of the decrease of elastic modulus. In view of our findings, it should be stated that a degree of damage in the investigated PMCCs should not be evaluated in line with a methodology using the elastic modulus as a descriptor. To evaluate a hydro-thermal fatigue of the tested polymer–ceramic composites, it seems that the strain work to fracture can be used.

## Figures and Tables

**Table 1:** Details about tested composites.

Parameter	Name of Materials		
	Filtek Z550 (Abbreviation—Z550)	Ex-nano(G)	Ex-Flow(G)
**Manufacturer**	3M ESPE (USA)	–	–
**Type**	Nanohybrid composite	Nano composite	Semi-liquid composite
**Matrix**	BIS-GMA, UDMA, BIS-EMA, PEGDMA, TEGDMA	BIS-GMA, UDMA, TEGDMA	BIS-GMA, UDMA, TEGDMA
**Filler**	SiO_2_ 20 nm, ZrO_2_/SiO_2_ 5–20 nm, (0.6–1.4 mm clusters)	The inorganic filler particles consist of barium aluminum bore glass and highly dispersed silicon dioxide.	The inorganic filler particles comprise silica, dental glass (strontium aluminum-boron-silicate glass)
**Content of Filler Molecules (wt.%)**	82%	82%	74%

**Table 2:** Descriptive statistics of the results obtained in the BFS test.

Quantity	F_BI_	dL (F_BI_)	F_Fract_	dL (F_Fract_)	W (F_BI_)	W(F_Fract_)	h
Unit	*N*	*mm*	*N*	*mm*	*Nmm*	*Nmm*	*mm*
Material	***Z550***						
	*n_TC_ = 0*						
$\bar{x}$	154.00	0.10	59.90	0.10	10.70	10.80	1.44
Std.dev.	24.60	0.00	9.50	0.00	3.72	3.73	0.065
ν	15.97	22.14	15.86	21.93	34.81	34.50	4.53
	*n_TC_ = 10,000*						
$\bar{x}$	129.00	0.10	49.00	0.10	8.21	8.51	1.31
Std.dev.	36.70	0.00	15.40	0.00	3.92	3.65	0.074
CV	28.42	26.01	31.34	21.25	47.76	42.86	5.66
Material	***Ex-nano(G)***						
	*n_TC_ = 0*						
$\bar{x}$	156.00	0.20	60.40	0.20	12.39	12.53	1.26
Std.dev.	10.60	0.00	4.17	0.00	1.30	1.30	0.057
CV	6.80	6.81	6.90	6.72	10.47	10.37	4.51
	*n_TC_ = 10,000*						
$\bar{x}$	93.90	0.10	36.50	0.10	5.13	5.19	1.31
Std.dev.	14.30	0.00	5.85	0.00	1.50	1.50	0.071
CV	15.23	14.10	16.03	13.92	29.23	28.94	5.42
Material	***Ex-flow(G)***						
	*n_TC_ = 0*						
$\bar{x}$	114.00	0.10	44.20	0.10	8.80	8.88	1.24
Std.dev.	19.00	0.00	7.22	0.00	2.75	2.75	0.055
CV	16.67	15.51	16.33	15.29	31.30	30.93	4.43
	*n_TC_ = 10,000*						
$\bar{x}$	81.30	0.10	31.80	0.10	4.41	4.45	1.24
Std.dev.	12.40	0.00	4.93	0.00	1.31	1.31	0.063
CV	15.28	17.29	15.51	17.06	29.77	29.49	5.08

**Table 3:** Descriptive statistics of the results obtained in the TFS test.

Quantity	E_f_	σ_fM_	ε_fM_	σ_fB_	ε_fB_	W_fM_	W_fB_
Unit	*MPa*	*MPa*	*%*	*MPa*	*%*	*Nmm*	*Nmm*
Material	***Z550***						
*n_TC_ = 0*							
$\bar{x}$	6730	71.2	1.1	62.9	1.1	4.75	4.76
Std.dev.	1120	16.8	0.3	16.8	0.3	2.24	2.25
CV	16.65	23.66	24.76	23.66	24.76	47.24	47.24
*n_TC_ = 10,000*							
$\bar{x}$	9990	59.4	0.7	53.0	0.7	2.36	2.37
Std.dev.	1700	20.6	0.3	14.4	0.3	1.51	1.52
CV	17.04	34.66	48.94	27.12	48.76	64.16	64.20
Material	**Ex-nano (G)**						
*n_TC_ = 0*							
$\bar{x}$	9910	99.5	1.1	78.4	1.1	6.41	6.43
Std.dev.	1010	14.9	0.2	8.97	0.2	2.01	2.01
CV	10.19	14.97	19.54	11.44	19.53	31.31	31.27
*n_TC_ = 10,000*							
$\bar{x}$	9030	44.4	0.5	44.4	0.5	1.19	1.19
Std.dev.	753	9.38	0.1	9.40	0.1	0.55	0.55
CV	8.34	21.11	23.45	21.18	23.36	46.21	46.08
Material	**Ex-flow (G)** (N = 15)						
*n_TC_ = 0*							
$\bar{x}$	9190	54.4	0.5	54.4	0.5	1.65	1.65
Std.dev.	517	9.86	0.1	9.86	0.1	0.6	0.6
CV	5.63	18.12	16.99	18.12	16.99	36.17	36.17
*n_TC_ = 10,000*							
$\bar{x}$	7980	21.5	0.2	21.5	0.2	0.30	0.30
Std.dev.	605	2.98	0.0	3.02	0.0	0.09	0.09
CV	7.58	13.85	21.87	14.08	21.46	30.64	30.04

## References

[1] Willems G., Lambrechts P., Braem M., Celis J.P., Vanherle G. (1992). A classification of dental composites according to their morphological and mechanical characteristics. Dent. Mater..

[2] Braem M.J., Davidson C.L., Lambrechts P., Vanherle G. (1994). In vitro flexural fatigue limits of dental composites. J. Biomed. Mater. Res..

[3] Madej M., Ozimina D., Cwanek J., Styp-Rekowski M. (2010). The analysis of tribological wear of polythene UHMW PE applied in biotribological systems. Tribologia.

[4] Będziński R. (2007). Experimental and numerical methods in biomechanics. Biocybern. Biomed. Eng..

[5] Pieniak D. (2018). Initiation and tolerance of macro-damage of first ply (fbf) in a process of damaging of hybrid multi-ply structures due to reinforcement architecture. Adv. Mater. Sci..

[6] Pieniak D., Niewczas A. (2012). Phenomenological evaluation of fatigue cracking of dental restorations under conditions of cyclic mechanical loads. Acta Bioeng. Biomech..

[7] Eftekhari M., Fatemi A. (2016). On the strengthening effect of increasing cycling frequency on fatigue behavior of some polymers and their composites: Experiments and modeling. Int. J. Fatigue.

[8] Kelly J.R. (1995). Perspectives on strength. Dent. Mater..

[9] Rodrigues S.A., Ferracane J.L., Bona A.D. (2008). Flexural strength and Weibull analysis of a microhybrid and a nanofill composite evaluated by 3- and 4-point bending tests. Dent. Mater..

[10] Bechtold J., dos Santos P.J., Anido-Anido A., di Hipolito V., Alonso R.C.B., D’Alpino P.H.P. (2012). Hardness, polymerization depth, and internal adaptation of Class II silorane composite restorations as a function of polymerization protocol. Eur. J. Dent..

[11] Asmussen E., Peutzfeldt A. (1998). Influence of UEDMA, BisGMA and TEGDMA on selected mechanical properties of experimental resin composites. Dent. Mater..

[12] Fu S.Y., Feng X.Q., Lauke B., Mai Y.W. (2008). Effects of particle size, particle/matrix interface adhesion and particle loading on mechanical properties of particulate-polymer composites. Compos. Part B Eng..

[13] Gurumurthy C.K., Kramer E.J., Hui C. (2001). Hydro-thermal fatigue of polymer interfaces. Hydro-thermal fatigue of polymer interfaces. Acta Mater..

[14] Jakowluk A. (1993). Procesy Pełzania i Zmęczenia w Materiałach.

[15] Hales R., Skelton R.P. (1983). Fatigue testing methods of elevated temperature. Fatigue at High Temperature.

[16] Lohbauer U., Belli R., Ferracene J.L. (2013). Factors involved in mechanical fatigue degradation of dental resin composites. J. Dent. Res..

[17] Milewski G., Będziński R. (2011). Biomechanika w stomatologii zachowawczej. Biomechanika, Tom XII.

[18] Kim M.H., Min S.H., Ferracane J., Lee I.B. (2010). Initial dynamic viscoelasticity change of composites during light curing. Dent. Mater..

[19] Ornaghi B.P., Meier M.M., Rosa V., Cesar P.F., Lohbauer U., Braga R.R. (2012). Subcritical crack growth and in vitro lifetime prediction of resin composites with different filler distributions. Dent. Mater..

[20] Ferracane J.L. (2006). Hygroscopic and hydrolytic effects in dental polymer networks. Dent. Mater..

[21] Finer Y., Santerre J.P. (2004). The influence of resin chemistry on a dental composite’s biodegradation. J. Biomed. Mater. Res. A.

[22] Janda R., Roulet J.F., Latta M., Ruttermann S. (2007). Water sorption and solubility of contemporary resin-based filling materials. J. Biomed. Mater. Res. Part B Appl. Biomater..

[23] Leibrock H., Degenhart M., Behr M., Rosentritt M., Handel G. (1999). In vitro study on the effect of thermo- and load-cycling on the bond strength of porcelain repair systems. J. Oral Rehabil..

[24] Akin H., Ozdemir A.K. (2013). Effect of corrosive environments and thermocycling on the attractive force of four types of dental magnetic attachments. J. Den. Sci..

[25] Morresi A.L., D’Amarioa M., Capogreco M., Gatto R., Marzo G., D’Arcangelo C., Monaco A. (2014). Thermal cycling for restorative materials: Does a standardized protocol exist in laboratory testing? A literature review. J. Mech. Behav. Biomed..

[26] Cavalcanti A.N., Mitsui F.H.O., Ambrosano G.M.B., Marchi G.M. (2007). Influence of adhesive systems and flowable composite lining on bond strength of class II restorations submitted to thermal and mechanical stresses. J. Biomed. Mater. Res. Part B Appl. Biomater..

[27] Walczak A., Niewczas A., Pieniak D., Gil L., Kozłowski E., Kordos P. (2018). Temporary Stability of Compressive Strength of Flow and Universal Type LC PMCCS Materials. Adv. Mat. Sci..

[28] Pieniak D., Walczak A., Niewczas A.M., Przystupa K. (2019). The effect of thermocycling on surface layer properties of light cured polymer matrix ceramic composites (PMCCs) used in sliding friction pair. Materials..

[29] Pieniak D., Kordos P. (2019). Analysis of the degree of hydro-thermal fatigue damage of the surface layer of polymer-ceramic composites intended for operation in a biotribological node. Tribologia.

[30] Gale M.S., Darvell B.W. (1999). Thermal cycling procedures for laboratory testing of dental restorations. J. Dent..

[31] Karbhari V.M., Strassler H. (2007). Effect of fiber architecture on flexural characteristics and fracture of fiber-reinforced dental composites. Dent. Mater..

[32] (2019). ISO 4049–Dentistry—Polymer-Based Restorative Materials.

[33] Hahnel S., Dowling A.H., El-Safty S., Fleming G.J.P. (2012). The influence of monomeric resin and filler characteristics on the performance of experimental resin-based composites (RNCs) derived from a commercial formulation. Dent. Mater..

[34] Walczak A., Pieniak D., Niewczas A., Niewczas A.M., Kordos P. (2015). Study of Ceramic-Polymer Composites Reliability Based on the Bending Strength Test. J. KONBiN.

[35] Niewczas A.M., Pieniak D., Ogrodnik P. (2012). Reliability analysis of strength of dental composites subjected to different photopolymerization procedures. Eksploat. Niezawodn..

[36] Palin W.M., Fleming G.J.P., Marquis P.M. (2005). The reliability of standardized flexure strength testing procedures for light-activated resin-based composite. Dent. Mater..

[37] St-Georges A.J., Swift E.J., Thompson J.Y., Heymann H.O. (2003). Irradiance effects on the mechanical properties of universal hybrid and flowable hybrid resin composites. Dent. Mater..

[38] (2015). ISO 6872 – Dentistry—Ceramic Materials.

[39] Braem M., Lambrechts P., Van Doren V., Vanherle G. (1986). The impact of composite structure on its elastic response. J. Dent. Res..

[40] Gonçalves F., Kawano Y., Braga R.R. (2010). Contraction stress related to composite inorganic content. Dent. Mater..

[41] Kim K.H., Ong J.L., Okuno O. (2002). The effect of filler loading and morphology on the mechanical properties of contemporary composites. J. Prosthet. Dent..

[42] Antunes P.V., Ramahlo A., Carrihlo E.V.P. (2014). Mechanical and wear behaviours of nano and microfilled polymeric composite: Effect of filler fraction and size. Mater. Des..

[43] Ikejima I., Nomoto R., McCabe J.F. (2002). Shear punch strength and flexural strength of model composites with varying filler volume fraction, particle size and silanization. Dent. Mater..

[44] Ivanisevic A., Lainovic T., Blazic L., Vilotic M. (2014). Influence of Light-curing Mode on the Mechanical Properties of Dental Resin Nanocomposites. Proc. Eng..

[45] Walker M.P., Haj-Ali R., Wang Y., Hunziker D., Williams K.B. (2006). Influence of environmental conditions on dental composite flexural properties. Dent. Mater..

[46] Adabo G.L., dos Santos Cruz C.A., Fonseca R.G., Vaz L.G. (2003). The volumetric fraction of inorganic particles and the flexural strength of composites for posterior teeth. J. Dent..

[47] Beun S., Glorieux T., Devaux J., Vreven J., Leloup G. (2007). Characterization of nanofilled compared to universal and microfilled composites. Dent. Mater..

[48] Ilie N., Hickel R. (2009). Investigations on mechanical behaviour of dental composites. Clin. Oral Investig..

[49] Leinfelder K.F., Bayne S.C., Swift E.J. (1990). Packable composites: Overview and technical considerations. J. Esthet. Dent..

[50] Heintze S.D., Zellweger G., Zappini G. (2007). The relationship between physical parameters and wear of dental composites. Wear.

[51] Shalaby W.S., Salz U. (2007). Polymers for Dental and Orthopedic Applications.

[52] Sideridou I., Karabela M.M., Vouvoudi E.C. (2011). Physical properties of current dental nanohybrid and nanofill light-cured resin composites. Dent. Mater..

[53] Mair L.H., Stolarski T.A., Vowles R.W., Lloyd C.H. (1996). Wear: Mechanisms, manifestations and measurement. Rep. Workshop J. Dent..

[54] Kawano F., Ohguri T., Ichikawa T., Matsumoto N. (2001). Influence of thermal cycles in water on flexural strength of laboratory-processed composite resin. J. Oral Rehabil..

[55] Weir M.D., Moreau J.L., Levine E.D., Strassler H.E., Chow L.C., Xu H.H.K. (2012). Nanocomposite containing CaF_2_ nanoparticles: Thermal cycling, wear and long-term water-aging. Dent. Mater..

[56] Wang J., Kochan O., Przystupa K., Su J. (2019). Information-Measuring System to Study the Thermocouple with Controlled Temperature Field. Meas. Sci. Rev..

[57] Moraes J.S., Sostena M.M.D.S., Grandini C.R., Cuppoletti J. (2011). The Glass Transition Temperature in Dental Composites. Ceramic and Polymeric Composites for Various Uses.

[58] Souza R.O., Ozcan M., Michida S.M., de Melo R.M., Pavanelli C.A., Bottino M.A., Soares L.E., Martin A.A. (2010). Conversion degree of indirect resin composites and effect of thermocycling o their physical properties. J. Prosthodont..

[59] Ulker M., Ozcan M., Sengun A., Ozer F., Belli S. (2010). Effect of artificial aging regimens on the performance of self etching adhesives. J. Biomed. Mater. Res. Part B Appl. Biomater..

[60] Seimenis I., Sarafianou A., Papadopoulou H., Papadopoulou T.R.J. (2006). Shear bond strength of three veneering resins to a Ni-Cr alloy using two bonding procedures. J. Oral Rehabil..

[61] Curtis A.R., Palina W.M., Fleming G.J.P., Shortall A.C.C., Marquis P.M. (2009). The mechanical properties of nanofilled resin-based composites: The impact of dry and wet cyclic pre-loading on bi-axial flexure strength. Dent. Mater..

[62] Yu B., Liu D., Liu F., He J. (2014). Preparation and characterization of light-cured dental resin without methacrylate monomers derived from Bisphenol A. Adv. Polym. Tech..

[63] Wang Y., Lee J.J., Lloyd I.K., Wilson O.C., Rosenblum M., Thompson V. (2007). High modulus nanopowder reinforced dimethacrylate matrix composites for dental cement applications. J. Biomed. Mater. Res. A..

[64] Soderholm K.J., Belov A. (2012). Fracture of dental materials. Applied Fracture Mechanics.

[65] Sideridou I., Achilias D.S., Kyrikou E. (2004). Thermal expansion characteristics of light-cureddental resins and resin composites. Biomaterials.

[66] Bełzowski A. (2002). Metoda oceny stopnia uszkodzenia kompozytów polimerowych. Kompozyty.

